# Supplementary material for: ALCAM: A Novel Surface Marker on EpCAMlow Circulating Tumor Cells
Source: Biomedicines. 2022 Aug 16;10(8):1983. doi: 10.3390/biomedicines10081983 (PMC9405826; doi:10.3390/biomedicines10081983)

Supplementary Figure S1. G-Banding karyotyping of CTC cell lines (i) CF49, (ii) CM61 and (iii) HM59. Metaphase images from at least 5 cells per CTC were taken.

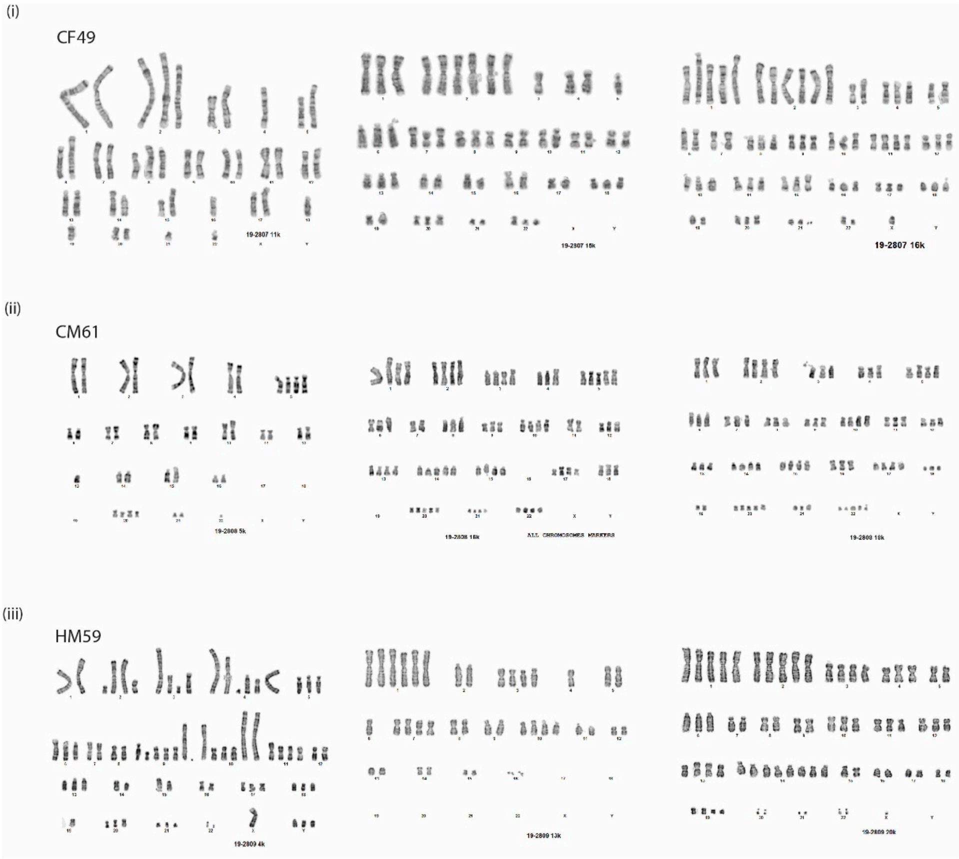

Supplementary Figure S2. TEM images of CM61 CTC cultured under (i) adherent and (ii) low attachment conditions.

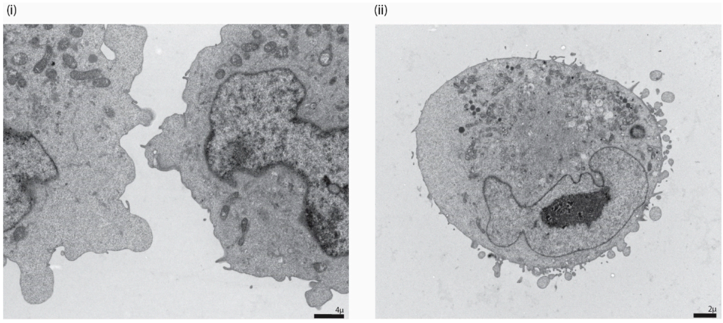

Supplement: Supplementary file 1 [file biomedicines-10-01983-s001.zip › biomedicines-1810144-supplementary.pdf]
